# Utility-driven assessment of anonymized data via clustering

**DOI:** 10.1038/s41597-022-01561-6

**Published:** 2022-07-30

**Authors:** Maria Eugénia Ferrão, Paula Prata, Paulo Fazendeiro

**Affiliations:** 1 Universidade da Beira Interior, Covilha, Portugal and CEMAPRE, Lisboa, Portugal; 2 Universidade da Beira Interior and Instituto de Telecomunicações (IT-UBI), Covilha, Portugal

**Keywords:** Education, Research data

## Abstract

In this study, clustering is conceived as an auxiliary tool to identify groups of special interest. This approach was applied to a real dataset concerning an entire Portuguese cohort of higher education Law students. Several anonymized clustering scenarios were compared against the original cluster solution. The clustering techniques were explored as data utility models in the context of data anonymization, using k-anonymity and (ε, δ﻿)-differential as privacy models. The purpose was to assess anonymized data utility by standard metrics, by the characteristics of the groups obtained, and the relative risk (a relevant metric in social sciences research). For a matter of self-containment, we present an overview of anonymization and clustering methods. We used a partitional clustering algorithm and analyzed several clustering validity indices to understand to what extent the data structure is preserved, or not, after data anonymization. The results suggest that for low dimensionality/cardinality datasets the anonymization procedure easily jeopardizes the clustering endeavor. In addition, there is evidence that relevant field-of-study estimates obtained from anonymized data are biased.

## Introduction

The increasing digitization of data in all the domains of our society makes privacy protection a challenge for data analysis. Administrative data primarily collected for official statistics offer great potential for secondary analyses such as quantitative based scientific research. Whenever the data controller delivers the data and makes such analyses possible, it is assumed that the individual rights set by the European General Data Protection Regulation (GDPR) are met^1^. This represents a great deal of statistical and data science challenges for the government sector^2,3^. In fact, GDPR stipulates that the data controller is responsible for proceeding to data anonymisation before delivering them to a third party^4^. There are two approaches for controlling the risk of statistical disclosure^5^. The first is based on data access control, e.g. restricting data access to “well-defined group of individuals […], under well-defined conditions […] in well-defined places”^5^; the second is based on statistical disclosure control (SDC) methods, meaning that the released data may have been changed or anonymized in order to reduce the risk of individual data disclosure. In a process of data anonymization the need to ensure the data usefulness is as important as ensuring the individual privacy.

Thus, data anonymization is an iterative process where after applying each privacy model and consequent assessment of the re-identification risk, the resultant anonymized data must be evaluated according to an utility model. The entire process should be repeated, until a reasonable balance is reached between minimizing the risk of re-identification and maintaining the maximum usefulness of the data^6,7^. Thus, the entire process consists of three main steps: (1) applying a privacy model; (2) quantifying the risk of disclosure or re-identification, and (3) assessing data utility.

Regarding privacy models, two main approaches have been followed: clustering algorithms based on the initial k-anonymity method^8^ and differential privacy (DP)^9^. Both privacy approaches have limitations since “privacy is a subjective concept, it varies from person to person, from time to time, even for the same person”^10^. The study presented in^11^ showed that it was possible to re-identify some individuals, by associating real names to their “k-anonymized” data records, from a previously de-identified publicly available dataset of applicants for the California Bar exam, who sat for the exam between 1977 and 2008. Their work draws attention to the fact that data controller does not usually convey any information on the protocols in use for data privacy. As demonstrated by^11^ such practice does not prevent against data re-identification. In addition, not publishing minimal information on anonymization protocols makes it impossible to study the trade-off between data utility and disclosure risk. Consequently, it does not contribute to reduce the lack of consensus on the balance between minimizing the risk of re-identification and maintaining the maximum usefulness of the data. For example, while^12^ refer that DP can require heavy data perturbation, leading to non-useful output^13^, refer that “one of the main advantages of this method regards managing the tradeoff between privacy and utility, finding the ideal value of which preserves data privacy and affects the model results at a controlled extent﻿”^13^. In fact, the literature offers a promise that in many situations DP provides higher levels of privacy while allowing extremely accurate statistics about the original database^14^.

The privacy level in k-anonymity implementations is related to k setting, and in DP it is related to ε setting. In general, data controller is the entity which holds the control for such regulatory scheme, which in turn tends to degrade data accuracy and impact utility of the data. Metrics to assess data utility may be classified according to resulting data accuracy, completeness, and consistency^12^. Standard metrics promptly available, such as the sum of squared error (SSE)^15^, the number of suppressed records or the non-uniform entropy^16^ do not take into account the specific loss for the substantive field of study. Thus, utility models and respective metrics may also account for the multivariate correlational loss in the anonymized data set, and for the misclassification error.

Regarding the use of administrative data for research and SDC methods, the aim of this study is twofold: firstly, we propose to adjust the utility model to the research question in the applied field of study, e.g. clustering analysis, as complementary to data utility quantified by standard metrics, no matter the substantive applied field of study; secondly, we intend to provide insight into the differences between anonymized and original datasets and debate its relevance for research purposes. As example, the methodological approach is applied in the field of higher education (HE) research. This is a field where there is a critical need to quantify the rates of degree completion and to diagnose the risk factors of not earning the degree on time. By definition, risk assessment “attempts to identify those cases in which a subsequent negative event […] is most likely to occur”^﻿17^. Specifically, clustering analysis is applied in order to timely identify and characterize groups of students at risk of not completing their degree on time within the scope of a programme for students’ success in HE^18^. For the purpose of assessing such risk, the relative risk^19^ is applied.

## Methods

### Privacy models

Direct identifier, also known as formal identifier^20^, is any variable or set of variables that directly makes possible the identification of an individual without additional information. Its value is structurally unique. Those variable values that are not structurally unique in a dataset but may be empirically unique, that is, in combination with other attributes (within the dataset or from other datasets) allowing for individual re-identification, are known as quasi-identifiers^20^. In addition, those variables whose content belongs “to the private domain of respondents who would not like them to be disclosed” are known as sensitive variables^20^. For example, data relating to religion, politics, health, etc. are considered sensitive under the EU’s data protection law. Thus, the first step of anonymization is to classify the attributes of the dataset as direct identifiers, quasi-identifiers, and/or sensitive, according to their characteristics. After that, direct identifiers are removed and quasi-identifiers may be recoded to guarantee that the data fulfils the privacy criteria. This may be achieved by the quasi-identifiers’ generalization, that is, by joining categories or classes of variables by changing the scale or order of magnitude. After defining possible generalization hierarchies, an anonymization algorithm should be used to find the minimal generalization that satisfies the desired privacy model.

The privacy model of k-anonymity was first proposed by Sweeney and Samarati as follows: ﻿“Let T(A1, …, An) be a table and QI be the quasi-identifier associated with it. T is said to satisfy k-anonymity w.r.t. QI if and only if each sequence of values in T[QI] appears at least k occurrences in T[QI]”^﻿8^. The set of k records is called an equivalence class. Several algorithms have been proposed for k-anonymity using suppression and generalization transformations^11,12^. Some variants of the k-anonymity model have been proposed to deal with sensitive attributes, that is, attributes that consist of sensitive person specific information which individuals are not willing to be linked with, and, if disclosed, could cause harm to data subjects. If a sensitive attribute has the same value in all the records of an equivalence class, it can lead to attribute disclosure. The ℓ-diversity^21^ and t-closeness^22^ are examples of models that improve from k-anonymity, protecting data against attribute disclosure. Sensitive attributes are out of the scope of this study.

DP emerged in 2006 as a concept^9,14^. Currently, there are several probabilistic based anonymization techniques, which change the data either by addition of random noise^2,23–25^ or by random sampling^26,27^. The latter is used for the purpose of this study.

The DP model was proposed in^9^ as follows: A randomized function R gives ε-DP if for all data sets D1 and D2 differing on at most one record, and all S ⊆ Range(R),

1 $$\Pr [R\left(D1\right)\in S]\le \exp \left(\varepsilon \right)\ast \Pr [R\left(D2\right)\in S]$$

According to that definition, informally speaking, given the result of a transformation, one cannot tell if a specific record was in the dataset. The probability of producing a result is almost the same with or without that record. Most of the proposals to implement DP are based on adding noise to build an anonymized dataset^25,28^. A relaxed version of DP is (ε, δ)-DP, that states that a randomized function R provides (ε, δ)-DP if Eq. (1) holds with a probability of at least 1-δ^29^. The SafePub anonymization algorithm starts by randomly sampling the dataset, and after that explores it using the generalizations hierarchies defined by the user. For each generalization scheme the algorithm suppresses the records that appear less than k times searching for an optimal solution. The value of k is derived from the parameters ε and δ. The final anonymized dataset satisfies k-anonymity and is (ε, δ)-DP as shown in^27^.

### Disclosure risk models

As the number of sensitive or quasi-identifying variables increases, so does the risk of disclosure^30^. Measuring the risk of disclosure implies modelling the adversary’s background knowledge and not assuming that the attacker just knows the quasi-identifiers^31^. An adversary can learn background knowledge about an individual, the target, from social networks, demographic statistics, and many other sources. Three common attacker profiles are usually considered to quantify the re-identification risk, leading to the prosecutor risk, the journalist risk, and the marketer risk. These three profiles are related to the attacker’s characteristics, like motivation, means, intent to cause harm, and background knowledge of the target. For instance, the knowledge about the inclusion of the target in the disclosed data is crucial. If the attacker knows whether the target is in the anonymized data, then the re-identification risk is called prosecutor risk^32,33^. The metric usually used is the proportion of unique records in the anonymized data. If the attacker does not or cannot know whether the target is in the anonymized data, then the risk is known as journalist risk^32^. In turn, the marketer risk is quantified by the average proportion of records in the anonymized file that would be correctly re-identified if the attacker tried to re-identify everyone in the data set^34^. The attacker tries to re-identify as many subjects as possible. Thus, the relationship between the three risks is as follows, prosecutor risk ≥ journalist risk ≥ marketer risk^32^.

As stated by^35^ “if we manage the risk of re-identifying an individual subject, then we also manage the risk of multiple subjects being re-identified”. The open source ARX, Data Anonymization Tool^6^, provides estimates for those three attack models^32^.

### Utility models and metrics

It is intuitively obvious that there is a loss of information when any anonymization mechanism is applied to a protected data set. The tradeoff between data privacy and data utility when applying privacy-preserving mechanisms has deserved vast developments at least since 1993^5,12,36–39^. Data utility models and metrics aim at quantifying the quality of the anonymized data for further analysis and modelling purposes. For example^40^, present and discuss a selection of more than 80 privacy metrics. We broadly followed^41^ whose review divides metrics into two groups: those more suitable for general analysis or those for specific analysis. The first group includes information loss metrics that are defined to measure the similarity between the original and the anonymized data. For the specific analysis purposes, the data utility is assessed by comparing the accuracy of a certain task using the original and the anonymized data. The key element for our choices was that from the point of view of the data analyst, a potentially preserved data quality for the subsequent analysis and respective inferences could be the output. Thus, we assess data utility using several metrics. The concept of entropy was proposed as an utility model for quantifying the loss of information^5^ and a variant known as Non-Uniform Entropy^42^ is commonly recommended for data anonymization in scientific works for categorical variables, and mean squared error (MSE) for continuous variables^16^. Furthermore, we used summary statistics such as records count that allow a certain number of queries^43^, the SSE^15^, and the relative risk in order to illustrate a relational data property relevant for the field of application.

### Clustering as an utility indicator

Clustering is the process of searching for a finite and discrete set of data structures (categories or clusters) within a finite, otherwise unlabelled, usually multivariate data set. Two distinct, but complementary, facets are enclosed in this unsupervised learning task^44^ the elicitation of a model of the overall structure of the data and the pursuit for a manageable representation of a collection of objects into homogeneous groups. A fundamental goal of clustering is to provide a meaningful insight on the structure of data, if such structure exists at all.

The partitional clustering algorithms attempt to directly decompose the data set into a collection of disjoint clusters. This partition is built during an iterative optimization process repeated until its associated cost function reaches a minimum (global or local). The cost function, also designed performance index or objective function, is a mathematical criterion expressing some desired features (emphasizing local or global structure of the data) of the resulting partition. Combining some heuristics with an adequate formulation of the objective function, it is possible to design an optimization process which can determine at least suboptimal partitions. The c-Means algorithm (also referred in the literature as k-Means or hard c-Means)^45^ is the best-known squared error-based example of such a process. For a given initialization of the c centroids the heuristic approach consists of two-step major iterations that follow from the first-order optimality conditions: first reassign all the points to their nearest cluster, thus updating the partition matrix, and then recompute the centroids of the newly assembled groups. This iterative procedure continues until a stopping criterion is achieved (usually until no reassignments happen).

The output of a clustering algorithm is a hypothesis on the summarization or explanation of data. As such, knowledge from the application field becomes of paramount importance. Experts are in the best position to evaluate the results of clustering and to select the suitable level of granularity that is required for a given task cf.^46^. Exploratory analysis using clustering involves a number of steps including selection and application of clustering algorithm; validation according to some cluster validity index; and a final, crucial, interpretation step^47^. This interpretation depends on the application field and relies on the existence of domain experts. The number of clusters c is the most important parameter for the standard partitional clustering algorithms. If c is equal to the unknown number of subgroups present in the data, there is a higher chance that the clustering process effectively reveals the underlying structure of the data. The effectiveness of this choice is verified by cluster validity analysis^48^. One possible way of performing a classical cluster validity analysis consists in running the clustering algorithm for different values of c, several times with different initializations. The validity of the obtained partitions is assessed by validity measures: the number c which optimizes one of these measures (or a combination of some of them) is chosen as the optimal one. In general, the objective is to seek for groups in data so that data in one group are similar to each other and are as different as possible from data in other groups.

In the context of privacy and utility preserving, we propose the cluster validity analysis as a complementary way to assess the utility of the anonymization model for the problems requiring exploratory data analysis solutions e.g.^49^. In this sense the anonymization can be seen as a denoising procedure that increases the probability of discarding small clusters with consequent prejudice on the correct identification of the underlying data structure. Moreover, as is stressed in^50^, there are cases where accurately identifying small-cluster information is more important than identifying large clusters. We argue that, by performing the cluster validity analysis on the anonymized dataset and the sequent comparison with the results obtained for the original dataset, the utility is only preserved whenever the underlying structure of the data does not change, hence allowing the correct identification of groups of interest for the study at hand.

### Relative risk as a metric for policy purposes

Consider the partition of individuals in the population into two disjoint groups: male and female students. For the thematic field of this work, relative risk, also known as risk ratio, is based upon the incidence rates of failing the degree on time in the group of male students and the group of female students. It is a metric of the association of failing the degree with the groups defined. In practice we estimate the relative risk (RR) via 2 × 2 contingency table (Table 1), such that

$$RR=\frac{a/\left(a+b\right)}{c/\left(c+d\right)}$$

**Table 1:** Contingency table for relative risk.

Gender	Degree	
	Not on time	On time
Male	a	b
Female	c	d

### Data safety and procedures

We used microdata of the administrative data known as “Registo de Alunos Inscritos e Diplomados do Ensino Superior” [Register of students enrolled in and graduated from higher education] (RAIDES) made available by the Directorate-General of Education and Science Statistics (DGEEC) under the protocol 5/2020 with the research centre CEMAPRE for data privacy protection. The protocol includes the identification of the research team and their research purpose for data access. One of the authors had access to two pseudonymized data files in the safe center in Lisbon, after having signed a data usage agreement. All files that came out of the safe center had been previously verified and authorized by the data protection officer. The procedure set for a research team to get access to RAIDES files is defined in concordance with the Portuguese law for personal data protection. To start the process, the principal researcher must send the electronic request through the form available at the following link https://www.dgeec.mec.pt/np4/pedido_dados.

The survey RAIDES is annually carried out within the scope of the National Statistical System which is mandatory. Data were collected by higher education institutions and exported in XML format to the DGEEC twice a year (January and April; December 31 and March 31 as time reference, respectively), throughout the “Plataforma de Recolha de Informação do Ensino Superior” [Platform of Data Collection in Higher Education] (PRIES). Regarding the two aforementioned files, the first refers to enrolments in a given academic year and the second refers to graduates in a given academic year. Data processing and anonymization procedures were conducted in the safe center, applying the methodology developed by the authors of this work. For the purpose of the study, students’ data enrolled in law undergraduate courses in the academic year 2013–14 and graduates’ data in the academic year 2016–17 were paired. Records of students who were not enrolled in their 1st year for the first time or whose access to HE was different from the national competition were not considered in our analyses. The following students’ attributes were considered: Degree conclusion on due time (DC, Yes/No); University entrance score (UES) that was standardised by using the national mean and standard deviation; Gender (1: female; 0: male); and Age at enrolment (in years).

### Participants

We considered students who entered undergraduate law programs by the general contingent in the 2013–14 academic year. The number of students involved was 1659, of which 43% earn their degree in due time. Distribution per gender was 65.8% female and 34.2% male; students’ age at enrolment had a mean of 18.59 (SD = 1.47) and a median of 18. Conditional distribution of degree completion given gender is presented in Table 2. The percentage of graduates was 47.9 in the female group and 33.7 in the male. Thus, the relative risk of not earning the degree in due time is 1.27 for males, compared to females. This suggests male students are 27% more likely than female ones to fail the degree conclusion on time.

**Table 2:** Conditional distribution 1st cycle degree completion given gender.

Gender	Degree not on time	Degree on time
Male	66.3	33.7
Female	52.1	47.9
Total	57.0	43.0

### Design of the study

Clustering was applied to the dataset described in the previous sections, and to four anonymized datasets. The first, DS1, was obtained by k-anonymization with k = 20; the remaining three were obtained by (ε, δ)-DP with fixed ε = 1, and δ = 0.01, δ = 0.001 and δ = 0.0001 for DS2, DS3 and DS4 respectively. The parameters k, ε, δ were chosen in such way that the re-identification risk is less than 5%.

The anonymization was performed with ARX (version 3.9.0). The parameters were defined as follows, suppression limit: 100%; utility measure: loss; aggregate function: sum; population size: 5000. The attributes classified as quasi-identifiers were Age and UES and for each of them a generalization hierarchy, where the values of the attribute may be transformed into intervals with decreasing precision over increasing levels of generalization, was defined. For the attribute Age, two levels of generalization were defined, at the first level, Age is transformed into the intervals [17,19[and [19, 54[, at the second level, Age is suppressed. The attribute UES was generalized using the standard deviation as the standardised scale unit, and the mean point of every interval was used in posterior data processing. The second level of UES generalization was suppression. The set of all possible combinations of generalizations levels for both attributes is explored by the anonymization algorithm. For each scheme, all records that do not comply with the privacy requirements are suppressed and the utility of the resulting dataset is assessed. At the end, the optimal solution, that is, the dataset with less loss of records is returned.

## Results

The main characteristics of the studied anonymized datasets, are presented in Table 3. The k value for DS1 was given by the user, but for datasets satisfying DP it was a result calculated by the SafePub algorithm. For these datasets, it can be seen that more restrictive values of δ lead to higher values of k. As expected, the number of records that remains after anonymization decreases when more restrictive privacy models are applied. It should be noted that in DP, the dataset is pre-processed by randomly sampling. That justifies the small number of records in DS2, although k = 19.

**Table 3:** Main characteristics of the studied datasets.

Dataset	Privacy model	K value	Number of records	Estimated risk
DS1	k-anonymity	20	1643	4.2%
DS2	(1, 0.01)-DP	19	892	3.8%
DS3	(1, 0.001)-DP	33	847	2.3%
DS4	(1, 0.0001)-DP	45	785	2.1%

For each dataset it is presented the journalist and prosecutor risk estimated by ARX (the value is the same for both attacker models). As can be seen, DP reduces the risk, but the number of remaining records is much smaller. Table 4 presents the number of different values per variable. It emphasizes that the overall data diversity is severely harmed.

**Table 4:** Number of different values per variable.

Dataset	Privacy model	Age	UES	DC	Gender
Original	—	16	483	2	2
DS1	k-anonymity	3	5	2	2
DS2	(1, 0.01)-DP	3	4	2	2
DS3	(1, 0.001)-DP	2	4	2	2
DS4	(1, 0.0001)-DP	2	4	2	2

A deeper look at the anonymized datasets shows that the generalization hierarchy for the attribute Age was never used. In DS1 and DS2 age values greater than 20 were suppressed. In addition, in DS3 and DS4 age values greater than 19 were removed. For the attribute UES, the first level of generalization was applied in all cases. Beyond generalization, in DS1 all records with UES = −1.5 were removed, and with DP records with UES = −1.5 and records with UES = 3.5 were removed.

The ARX tool supports some general-purpose utility models that can be used when it is unknown how the output data will be used. Table 5 shows the values obtained for the non-uniform entropy and for the sum of square errors when applied to the quasi-identifiers of each anonymized dataset. The non-uniform entropy compares the frequencies of attributes values in the transformed dataset with the according frequencies in the input dataset^16^. The sum of squared errors computes the sum of squares of attribute distances between records in the original data set and their versions in the anonymized data set^15^. According to ARX documentation, the values have been scaled and normalized into the range [0, 1] where 0 represents a dataset from which all information has been removed and 1 represents the unmodified input dataset. Consequently, higher values returned for the quality models are always better^6^.

**Table 5:** Quality models (non-uniform entropy and square error) for the quasi-identifiers.

Dataset	N-u. entropy	Squared error	N-u. entropy	Squared error
	Age	Age	UES	UES
DS1	86.04%	93.85%	22.60%	87.31%
DS2	81.11%	91.35%	22.65%	83.34%
DS3	72.57%	86.91%	19.88%	77.17%
DS4	72.93%	83.71%	20.01%	74.97%

As can be seen from Table 5, the loss of information increases when more restrictive privacy models are applied. As expected, the loss of information in the generalized attribute, UES, is higher than in attribute Age.

**Fig. 1:**
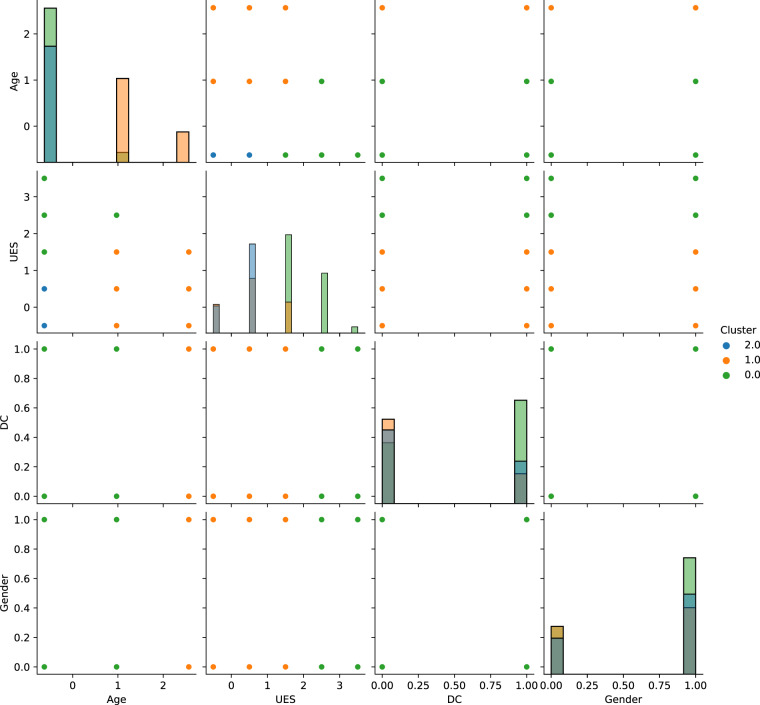
Anonymized dataset (k-anonymity) grouped in 3 clusters.

Figure 1 shows the data distribution between pairs of variables after anonymization (for DS1) assuming the formation of 3 different clusters. The number of distinct points in the dataset is very small - being even lesser for the remaining anonymized datasets due to the reduction of the discrete number of values per variable, see Table 4.

Regarding the data utility for the clustering problem at hand, we performed twenty independent runs of the hard c-means algorithm, each one with c ranging from 2 to 40 clusters. For the assessment of the quality of each partition in this work we choose three indices commonly used in practical applications: Silhouette, Davis-Bouldin and Calinski-Harabasz, cf.^51^. Broadly speaking, these indices estimate the clusters’ cohesion and the clusters’ separation, combining them to produce a quality measure. For Silhouette and Calinski-Harabasz the best partitions correspond to higher values whereas for Davis-Bouldin lower values are better.

**Fig. 2:**
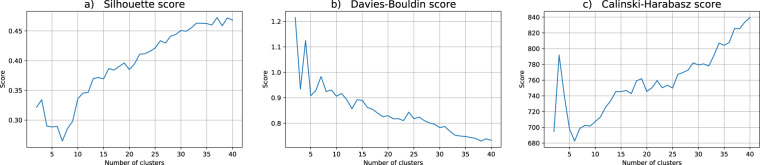
Cluster validity indices for the original dataset. The putative number of clusters range from 2 to 40. The depicted validity indices are (**a**) Silhouette, (**b**) Davis-Bouldin and (**c**) Calinski-Harabasz scores.

Figure 2 presents the mean values of the computed cluster validity indices for the original dataset. The cluster validity analysis seems to indicate that 3 clusters result in a good data partition (please notice a local maximum for Silhouette and Calinski-Harabasz metrics, and corresponding local minimum for Davis-Bouldin score). As a matter of fact, one of the resulting 3 clusters is very interesting in the sense that in the original data it allows to identify a particular Age range where the students are prone to take more time to finish the course than the strictly necessary.

**Fig. 3:**
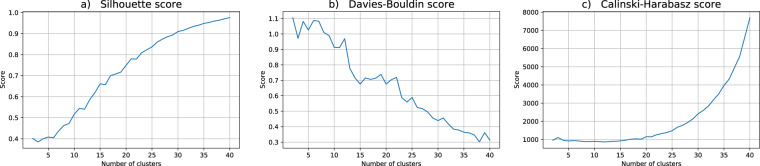
Cluster validity indices for the k-anonymity dataset (DS1). The putative number of clusters range from 2 to 40. The depicted validity indices are (**a**) Silhouette, (**b**) Davis-Bouldin and (**c**) Calinski-Harabasz scores.

Figure 3 depicts the cluster validity indices for the k-anonymity dataset (DS1). As can be seen in the graphs for Silhouette and Calinski-Harabasz, 3 is no longer suggested as a possible good choice for the right number of clusters. Moreover, the coherence between the indices behaviors is absent and the previous insight regarding the risk group is entirely lost when the anonymized data distribution is scrutinized (see also Fig. 1).

**Fig. 4:**
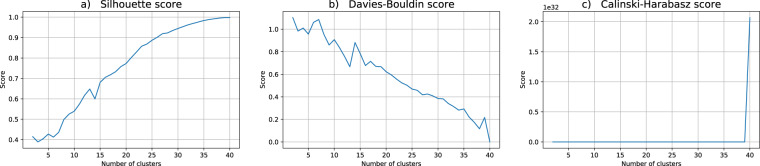
Cluster validity indices for the (1, 0.01)-DP dataset (DS2). The putative number of clusters range from 2 to 40. The depicted validity indices are (**a**) Silhouette, (**b**) Davis-Bouldin and (**c**) Calinski-Harabasz scores.

**Fig. 5:**
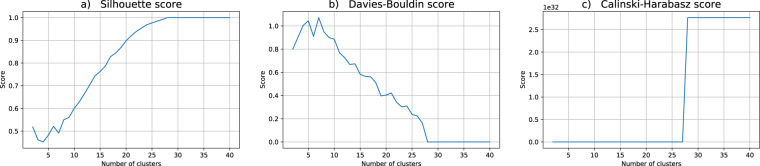
Cluster validity indices for the (1, 0.001)-DP dataset (DS3). The putative number of clusters range from 2 to 40. The depicted validity indices are (**a**) Silhouette, (**b**) Davis-Bouldin and (**c**) Calinski-Harabasz scores.

**Fig. 6:**
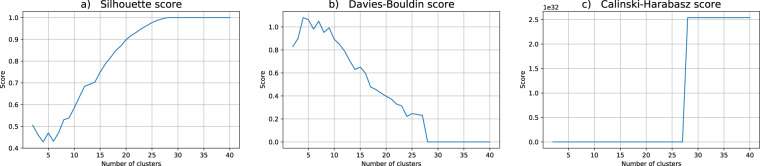
Cluster validity indices for the (1, 0.0001)-DP dataset (DS4). The putative number of clusters range from 2 to 40. The depicted validity indices are (**a**) Silhouette, (**b**) Davis-Bouldin and (**c**) Calinski-Harabasz scores.

Figures 4–6 show the behavior of the mean values of the computed cluster validity indices for the datasets DS2, DS3 and DS4 respectively. Those were obtained by (ε, δ)-DP keeping ε = 1, and varying δ = 0.01 (DS2), δ = 0.001 (DS3) and δ = 0.0001 (DS4). From the clustering validity point of view, the overall structure of data does not find correspondence with the original data. Besides that, Figs. 5 and 6 show that for DS3 and DS4 it is not possible to form more than 28 clusters since this is the maximum number of distinct points in the corresponding anonymized datasets. Once again, the right number of clusters that could be chosen with the support of the validity indices is different for different datasets which indicates that these are structurally diverse.

**Fig. 7:**
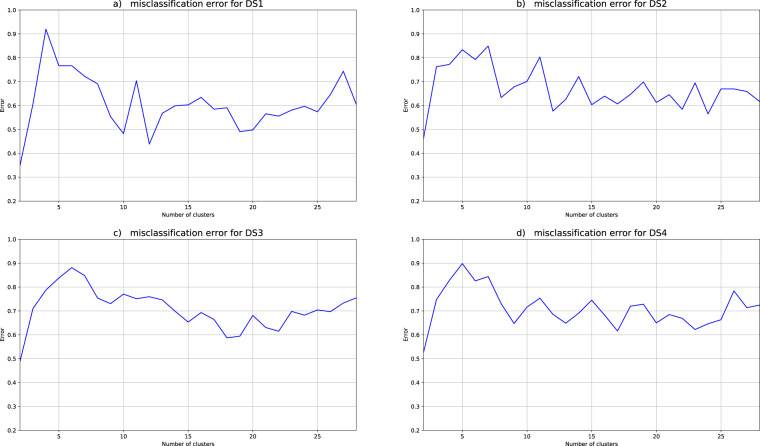
Misclassification Error per number of clusters. The putative number of clusters range from 2 to 28. Graph (**a**) shows the mean values for DS1, (**b**) presents the mean values for DS2 whereas (**c**) and (**d**) depict the same metric for DS3 and DS4 respectively.

Figure 7 presents the misclassification error of the anonymized datasets considering as the ground truth the clustering on the original data. The overall absolute error is bigger for the (ε, δ)-DP than for the k-anonymity scheme. Applying the (ε, δ)-DP scheme the overall absolute error increases as δ decreases.

Contingency tables obtained with DS1, DS2, DS3, and DS4 are presented in Table 6. As above mentioned, the true relative risk of male students not earning the degree on time is 1.27. Its point estimate computed from the anonymized data is 1.29, 1.29, 1.34, 1.32 for DS1, DS2, DS3, DS4, respectively, suggesting an upward bias.

**Table 6:** Contingency tables for relative risk.

Gender	Degree (DS1)		Degree (DS2)		Degree (DS3)		Degree (DS4)	
	Not on time	On time	Not on time	On time	Not on time	On time	Not on time	On time
Male	336	179	217	112	231	109	222	110
Female	520	508	336	321	337	329	314	306

## Discussion

It is well-known that standard data utility metrics often fail to properly quantify the accuracy and utility of anonymized data. This study compares the utility of a real dataset with the utility of four anonymized datasets obtained by different privacy models. Besides of providing a clear understanding of how anonymization may impact data usefulness, we argue for the relevance of taking into account the specific purpose of anonymization, and a novel purpose-oriented data utility model was applied. It is based on the application of an unsupervised clustering method (that can be tailored to the specificities of the available data) complemented with a clustering validity analysis - making use of indices that seek to accurately represent the desirable characteristics of the groups being formed (Silhouette, Davis-Bouldin and Calinski-Harabasz scores are just some of the possibilities). The model was applied to real and anonymized data collected from the field of HE survey and research. The anonymization process was based on k-anonymity and (ε,δ)-DP models. Our results show how powerful the tool of cluster validity analysis can be for timely identifying and characterizing groups of students at risk of not earning the degree on time. However, the results also suggest that, when applied to anonymized data, its power vanishes due to the loss of multivariate structure and diversity in the resulting dataset. Moreover, our findings suggest that relevant field-of-study estimates, such as the relative risk, obtained from anonymized data are biased. In a nutshell, when working with low dimensionality datasets, as the one used in this work (less than two thousand records and a small number of quasi-identifiers), no matter the method of anonymization, k-anonymity or DP, the results obtained suggest that the replacement of original data by their anonymized versions may jeopardize the proper data analysis, the data-based inferences or deductions and even the conclusions of the scientific research. Future work to understand the impact of anonymization when working with larger datasets should be done. It is hypothesized that having more records may result in greater data structure resilience, but, on the other hand, having more quasi-identifiers increases complexity and raises performance issues.

## Data Availability

The original data, RAIDES files, are protected by the GDPR. The consent for their use in the data safe center in Lisbon may be obtained by accredited researchers (https://www.dgeec.mec.pt/np4/pedido_dados). The anonymized datasets are publicly available in the Open Science Framework repository^52^.

## Code availability

The source code of the anonymization tasks is publicly available as open source software^6^. The clustering and cluster validity methods were based on the open source scikit-learn Python library implementation^53^. The clustering & validity pipeline source code is publicly available at https://github.com/Farmerinpt/clustering-anonymization-utility.
